# 2338. Robust and Persistent B Cell Responses Following Third Dose SARS-CoV-2 Vaccine Determine Protection from COVID-19

**DOI:** 10.1093/ofid/ofad500.1960

**Published:** 2023-11-27

**Authors:** Joanne Byrne, Lili Gu, Alejandro García-León, Colette Marie Gaillard, Julen Tomás-Cortázar, Riya Negi, Dana Alalwan, Gurvin Saini, Sean Donohue, Bearach Reynolds, Grace Kenny, Kelly Leamy, Tessa O’Gorman, Cathal O’Broin, Stefano Savinelli, Eoin R Feeney, Obada Yousif, Aoife Cotter, Eoghan de Barra, Corinna Sadlier, Alan Landay, Peter Doran, Rebecca J Cox, Ole Olesen, Jean-Daniel Lelièvre, Oliver A Cornely, Virginie Gautier, Patrick Mallon

**Affiliations:** Centre for Experimental Pathogen Host Research, University College Dublin, Ireland, Dublin, Ireland; 1Centre for Experimental Pathogen Host Research (CEPHR), University College Dublin, Belfield, Dublin 4, Ireland, Dublin, Dublin, Ireland; Centre for Experimental Pathogen Host Research (CEPHR), University College Dublin, Belfield, Dublin 4, Ireland, Dublin, Dublin, Ireland; 1Centre for Experimental Pathogen Host Research (CEPHR), University College Dublin, Belfield, Dublin 4, Ireland, Dublin, Dublin, Ireland; Centre for Experimental Pathogen Host Research (CEPHR), University College Dublin, Belfield, Dublin 4, Ireland, Dublin, Dublin, Ireland; 1Centre for Experimental Pathogen Host Research (CEPHR), University College Dublin, Belfield, Dublin 4, Ireland, Dublin, Dublin, Ireland; Centre for Experimental Pathogen Host Research (CEPHR), University College Dublin, Belfield, Dublin, Ireland; 1Centre for Experimental Pathogen Host Research (CEPHR), University College Dublin, Belfield, Dublin 4, Ireland, Dublin, Dublin, Ireland; St Vincent’s University Hospital, Elm Park, Dublin 4, Ireland, Dublin, Dublin, Ireland; 1Centre for Experimental Pathogen Host Research (CEPHR), University College Dublin, Belfield, Dublin 4, Ireland 2Department of Infectious Diseases, St Vincent’s University Hospital, Elm Park, Dublin 4, Ireland, Dublin, Dublin, Ireland; 1Centre for Experimental Pathogen Host Research (CEPHR), University College Dublin, Belfield, Dublin 4, Ireland 2Department of Infectious Diseases, St Vincent’s University Hospital, Elm Park, Dublin 4, Ireland, Dublin, Dublin, Ireland; 3School of Medicine, University College Dublin, Belfield, Dublin 4, Ireland5Department of Infectious Diseases, Mater Misericordiae University Hospital, Eccles St, Dublin 7, Ireland, Dublin, Dublin, Ireland; 1Centre for Experimental Pathogen Host Research (CEPHR), University College Dublin, Belfield, Dublin 4, Ireland5Department of Infectious Diseases, Mater Misericordiae University Hospital, Eccles St, Dublin 7, Ireland, Dublin, Dublin, Ireland; 1Centre for Experimental Pathogen Host Research (CEPHR), University College Dublin, Belfield, Dublin 4, Ireland 2Department of Infectious Diseases, St Vincent’s University Hospital, Elm Park, Dublin 4, Ireland, Dublin, Dublin, Ireland; Centre for Experimental Pathogen Host Research (CEPHR), University College Dublin, Belfield, Dublin 4, Ireland, Department of Infectious Diseases, St Vincent’s University Hospital, Elm Park, Dublin 4, Ireland, Dublin, Dublin, Ireland; Centre for Experimental Pathogen Host Research (CEPHR), University College Dublin, Belfield, Dublin 4, Ireland, Department of Infectious Diseases, St Vincent’s University Hospital, Elm Park, Dublin 4, Ireland, Dublin, Dublin, Ireland; 4Endocrinology Department, Wexford General Hospital, Carricklawn, Wexford, Ireland, Wexford, Wexford, Ireland; 1Centre for Experimental Pathogen Host Research (CEPHR), University College Dublin, Belfield, Dublin 4, Ireland5Department of Infectious Diseases, Mater Misericordiae University Hospital, Eccles St, Dublin 7, Ireland, Dublin, Dublin, Ireland; 6Department of Infectious Diseases, Beaumont Hospital, Beaumont, Dublin 9, Ireland7Department of International Health and Tropical Medicine, Royal College of Surgeons in Ireland, Dublin, Ireland, Dublin, Dublin, Ireland; Department of Infectious Diseases Cork University Hospital, Cork, Cork, Ireland; 9Department of Internal Medicine, Rush University, Chicago, Il, USA, Chicago, Illinois; 3School of Medicine, University College Dublin, Belfield, Dublin 4, Ireland, Dublin, Dublin, Ireland; Department of Clinical Science, University of Bergen, Norway, Bergen, Hordaland, Norway; European Vaccine Initiative, Heidelberg, Germany, Heidelberg, Baden-Wurttemberg, Germany; CHU Henri Mondor, Creteil, Ile-de-France, France; University of Cologne, Cologne, Germany, Cologne, Nordrhein-Westfalen, Germany; 1Centre for Experimental Pathogen Host Research (CEPHR), University College Dublin, Belfield, Dublin 4, Ireland, Dublin, Dublin, Ireland; University College Dublin, Dublin, Dublin, Ireland

## Abstract

**Background:**

The immunological memory to SARS-CoV-2 (SCV-2) vaccination has multiple components, with robust antibody (Ab) and B cell responses demonstrated post vaccination. The extent and persistence of T cell responses to vaccination remains unclear. We explored SCV-2 specific Ab, B cell and T cell responses to 3^rd^ dose vaccine and their relationship to incident COVID-19.

**Methods:**

In plasma from adults enrolled in a multicentre prospective cohort, sampled before, 14 days and 10 months post 3^rd^ dose vaccine (BNT162b2) we measured anti-SCV-2 receptor binding domain (RBD) Ab by electrochemiluminescence assays. Subjects reported incident COVID-19 that occurred post vaccination. In a subanalysis, we assessed SCV-2-specific plasma cell, memory B cell and T cell responses in peripheral blood mononuclear cells after stimulation with wild type (WT) RBD, WT full Spike antigen, Omicron RBD and Omicron S1 antigens by ELISpot (Mabtech ELISpot, Sweden, Fig 1). We compared between-group differences in immunological outcomes by incident infection by Wilcoxon signed rank or Mann–Whitney U tests. Data are median (IQR) unless specified.

**Figure:**
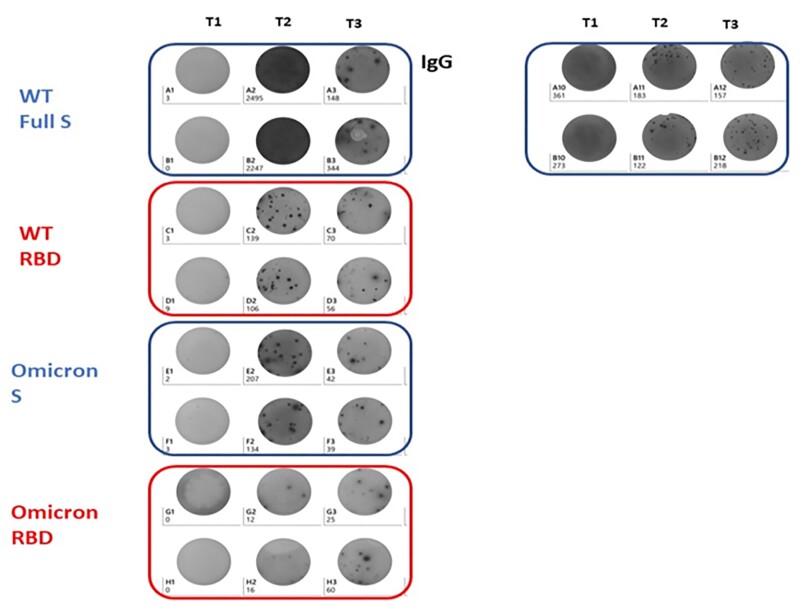

**Results:**

Of 132 subjects (age 43 [32-50], 81% female (Table 1), 47 (36%) reported incident SCV-2 infection at 18 (16-21) weeks post 3^rd^ vaccine. 76 subjects contributed to the cellular immunity subanalysis [23 of whom provided additional samples 10 months post vaccine (Table 1)]. RBD titres and B cell responses increased significantly 2 weeks post 3^rd^ vaccine (Fig 2, *p< 0.001*), with RBD titres and WT-specific memory B cell responses remaining significantly higher than pre-booster vaccine levels at 10 months (Fig 2, *p*< 0.001). In contrast, there was no significant difference in T cell responses at two weeks or 10 months post 3^rd^ dose vaccine (Fig 2). There was no difference in 2-week post vaccine RBD or T cell responses in those with and without incident SCV-2 infection. However, those with incident infection had significantly lower WT RBD-specific plasma and memory B cell levels (Table 2, all *p*< 0.05).

**Figure:**
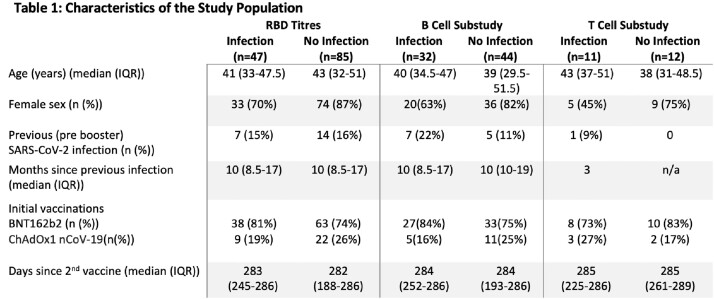

**Figure:**
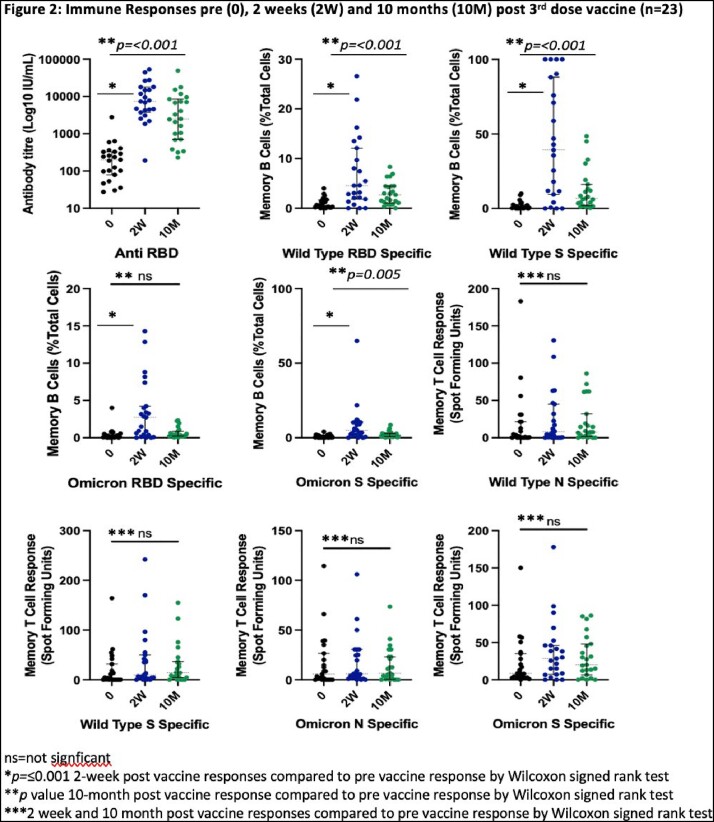

**Figure:**
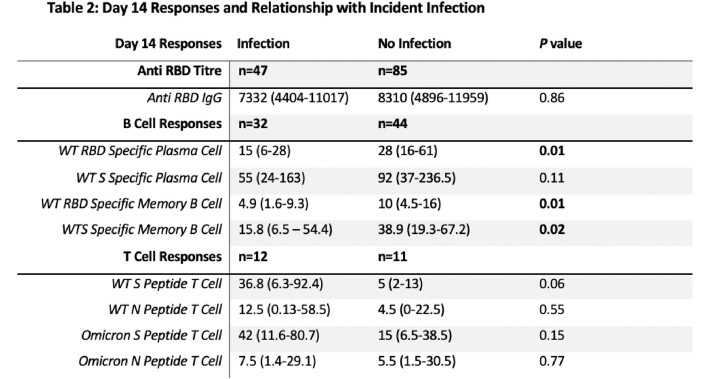

**Conclusion:**

3^rd^ dose vaccination induced robust antibody and B cell responses which persist at 10 months, but not T cell responses. Higher memory B cell responses post vaccination, rather than circulating antibody titres or T cells, are associated with protection from subsequent infection.

**Disclosures:**

**Oliver A. Cornely, MD PhD**, DZIF: Advisor/Consultant|DZIF: Board Member|DZIF: Grant/Research Support|DZIF: Honoraria|DZIF: Stocks/Bonds

